# Biomechanical Features of Backstroke to Breaststroke Transition Techniques in Age-Group Swimmers

**DOI:** 10.3389/fspor.2022.802967

**Published:** 2022-03-10

**Authors:** Phornpot Chainok, Karla de Jesus, Luis Mourão, Pedro Filipe Pereira Fonseca, Rodrigo Zacca, Ricardo J. Fernandes, João Paulo Vilas-Boas

**Affiliations:** ^1^Faculty of Sport Science, Burapha University, Mueang, Thailand; ^2^Centre of Research, Education, Innovation and Intervention in Sport (CIFI_2_D), Faculty of Sport, University of Porto, Porto, Portugal; ^3^Porto Biomechanics Laboratory (LABIOMEP-UP), University of Porto, Porto, Portugal; ^4^Faculty of Physical Education and Physiotherapy, Federal University of Amazonas, Manaus, Brazil; ^5^Superior Institute of Engineering of Porto, Polytechnic Institute of Porto, Porto, Portugal; ^6^Research Center in Physical Activity, Health and Leisure (CIAFEL), Faculty of Sports, University of Porto (FADEUP), Porto, Portugal; ^7^Laboratory for Integrative and Translational Research in Population Health (ITR), Porto, Portugal

**Keywords:** Exercise, aquatic locomotion, swimming, biomechanics, motion capture, force plate, hydrodynamics, performance

## Abstract

This study aimed to identify the biomechanical features of backstroke to breaststroke transition techniques (*open, somersault, bucket*, and *crossover*) in age-group swimmers. Eighteen preadolescent swimmers (12.2 ± 0.4 years old and 3–4 Tanner stages) underwent 4 weeks of systematic contextual interference training, comprising 16 sessions (40 min·session^−1^). Soon after, experimental testing was conducted where swimmers randomly performed 12 × 15 m maximal turns (composed of 7.5 m turn-in and 7.5 m turn-out of the wall segments), three in each transition technique. Kinematical, kinetic, and hydrodynamic variables were assessed with a dual-media motion capture system (12 land and 11 underwater cameras), triaxial underwater force plates, and inverse dynamics. Variables were grouped in turn-in (approach and rotation) and turn-out (wall contact, gliding, and pull-out) phases, with factor analysis used to select the variables entering on multiple regressions. For the turn-in phase, 86, 77, 89, and 87% of the variance for *open, somersault, bucket*, and *crossover* turning techniques, respectively, was accounted by the 7.5 and 2.5 m times, mean stroke length, and rotation time. For the turn-out phase, first gliding distance and time, second gliding depth, turn-out time, and dominating peak_Z push-off force accounted for 93% in open turn, while wall contact time, first gliding distance, breakout distance and time, turn-out time, dominating peak_Y push-off force, and second gliding drag coefficient accounted for 92% in a somersault turn. The foot plant index, push-off velocity, second gliding distance, and turn-out time accounted for 92% in bucket turn while breakout and turn-out time, non-dominating peak_Y and peak_Z push-off force, first and second gliding drag force and second gliding drag coefficient accounted for 90% in crossover turn, respectively. The findings in this study were novel and provided relevant biomechanical contribution, focusing on the key kinematic–temporal determinant during turn-in, rotation, and push-off efficacy, and the kinetic and hydrodynamic during turn-out, which would lead to improved backstroke to breaststroke transition techniques in 11–13 years-old age-group swimmers.

## Introduction

Performing fast and skilled turning actions, and start and swim phases, is fundamental for improving competitive swimming performance (Arellano et al., 1994; McGibbon et al., 2018; Zacca et al., 2020a). However, conclusive information on the 200- and 400-m individual medley events, in which butterfly, backstroke, breaststroke, and freestyle swim in this order, is limited. Therefore, extensive research is required to identify the key biomechanical variables and their respective contributions to each transition technique (Chainok et al., 2021).

Among the medley turns, there are four well-described backstroke to breaststroke transition techniques (the *open*, the *somersault*, the *bucket*, and the *crossover*), which are very complex movements (i.e., performed in different planes and axes). In addition, swimmers need to comply with the FINA rules, i.e., touch the wall while on their back, maintain the shoulders at or past the vertical direction toward the breast when leaving the wall, and assume a ventral gliding position prior to the first breaststroke upper limbs action. Studies on the backstroke to breaststroke transition techniques are scarce, lacking scientific and practical validation of the specific determinant factors that play a vital role in gaining the advantage in each backstroke to breaststroke transition techniques.

Key biomechanical variables related to swimming turn performance have been studied using temporal, kinematic (Blanksby et al., 2004; Araujo et al., 2010; Pereira et al., 2015), kinetic (Prins and Patz, 2006; Pereira et al., 2015; Chainok et al., 2021), and hydrodynamic data (Benjanuvatra et al., 2001; Vilas-Boas et al., 2010; Chainok et al., 2021), but no study has examined the biomechanical determinants for optimal backstroke to breaststroke transition performance. Knowing that this information is a key factor for coaches when planning their specific training activities, we aimed to identify the key biomechanical variables that affect the performance in the four backstroke to breaststroke transition techniques in age-group swimmers. It was hypothesized that the 15 m turning time performance is described by combining contributions from the turn-in and turn-out phases, and different combinations of feature variables depending on the chosen backstroke to breaststroke transition technique.

## Materials and Methods

### Subjects

A total of 18 age-group swimmers, nine boys and nine girls, from the 11–13 years old age group of a competitive swimming club, volunteered to participate in the current study. Boys and girls characteristics were (respectively): 12.5 ± 0.5 vs. 11.6 ± 0.5 years old, 48.7 ± 12.4 vs. 46.7 ± 10.8 kg of body mass, 1.59 ± 0.14 vs. 1.52 ± 0.07 m of height, 14.8 ± 5.1 vs. 21.8 ± 7.10% of fat mass, 3–4 Tanner stages (Zacca et al., 2020b) and 59 ± 9 vs. 55 ± 12% of 200 m individual medley best performances of the 2018 short-course World Junior Record. Swimmers parents were informed about the benefits and risks of participating before they were asked to sign an informed consent form (approved by the ethics board of the local university—CEFADE 08.2014) in agreement with the Declaration of Helsinki.

### Procedures

**Figure 1:**
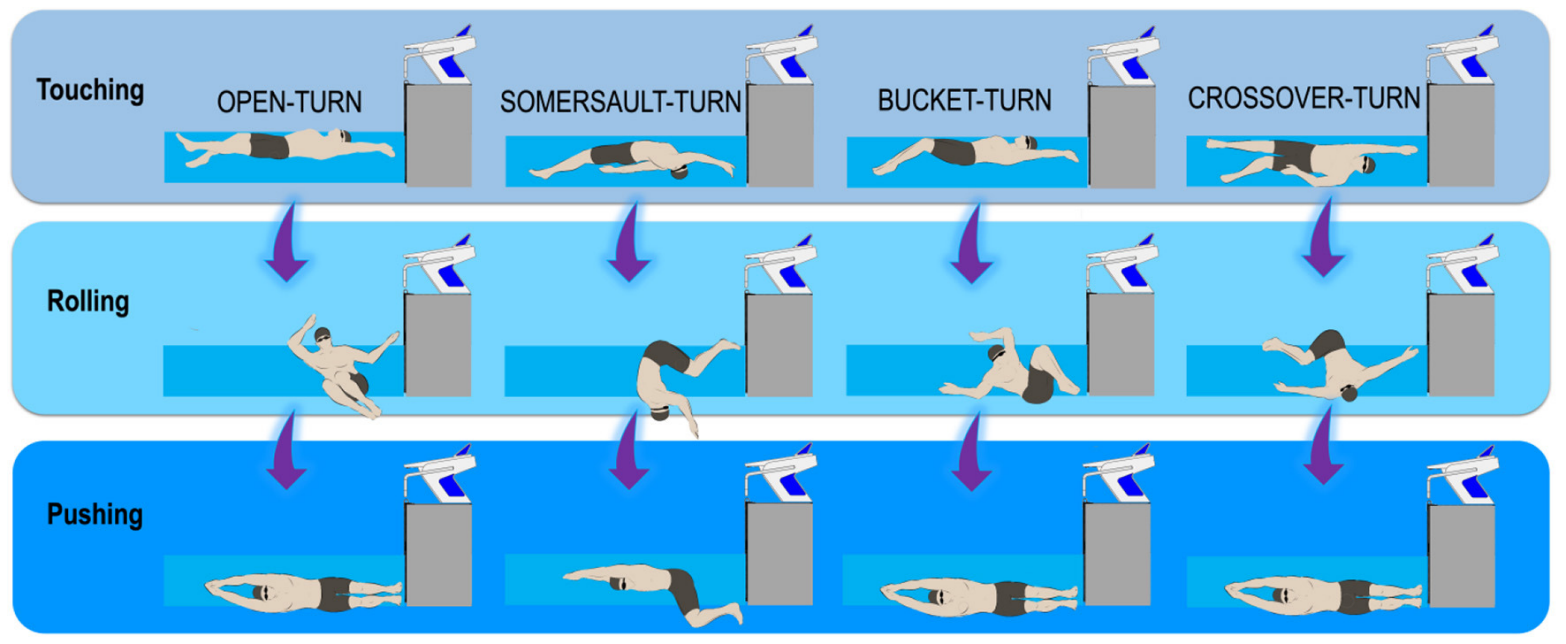
Backstroke to breaststroke turning techniques are distinguished by the different body orientations of the swimmers throughout the touching, rolling and pushing-off phases.

Four backstroke-to-breaststroke transition techniques were identified (FINA rules; https://www.fina.org/, see Figure 1). Prior to the experiments, swimmers answered a questionnaire about their backstroke to breaststroke transition techniques preferences, with 18 selecting the open technique and only two the somersault. The experimental protocol took place in a 25-m (1.90 m deep) indoor pool with ~27 and ~26°C of water and air temperatures (respectively) and 59% relative humidity. Age-group swimmers joined 16 practice sessions throughout a 4-week training program (see details in Chainok et al., 2021) performing variants of the same task with structured increases in contextual interference (Porter and Magill, 2010). Contextual interference can be defined as the interference in performance and learning that arises from practicing one task in the context of other tasks (Schmidt and Lee, 2005; Porter and Magill, 2010). The 16 practice sessions were part of the regular training sessions, with the turning practice occurring during the last 40 min of every session. Two experienced coaches conducted all practice sessions and specific coaching feedback based on mechanical factors to ensure consistency in coaching techniques, proper familiarization (Galbraith et al., 2008; de Jesus et al., 2016). All the participants followed a scheduled program from the 1st to the 16th practice session program (see details in Chainok et al., 2021). At the end of the intervention period, swimmers were invited for an evaluation session. Thus, after a 400-m moderate-intensity warm-up including some elements of backstroke to breaststroke transition techniques (Figure 1), swimmers were invited to perform 12 × 15 m maximal turns (composed of 7.5 m turn-in and 7.5 m turn-out of the wall segments). Each swimmer completed three attempts of each backstroke to breaststroke transition technique (randomized order), with a 3 min rest interval between trials (see details in Chainok et al., 2021).

**Figure 2:**
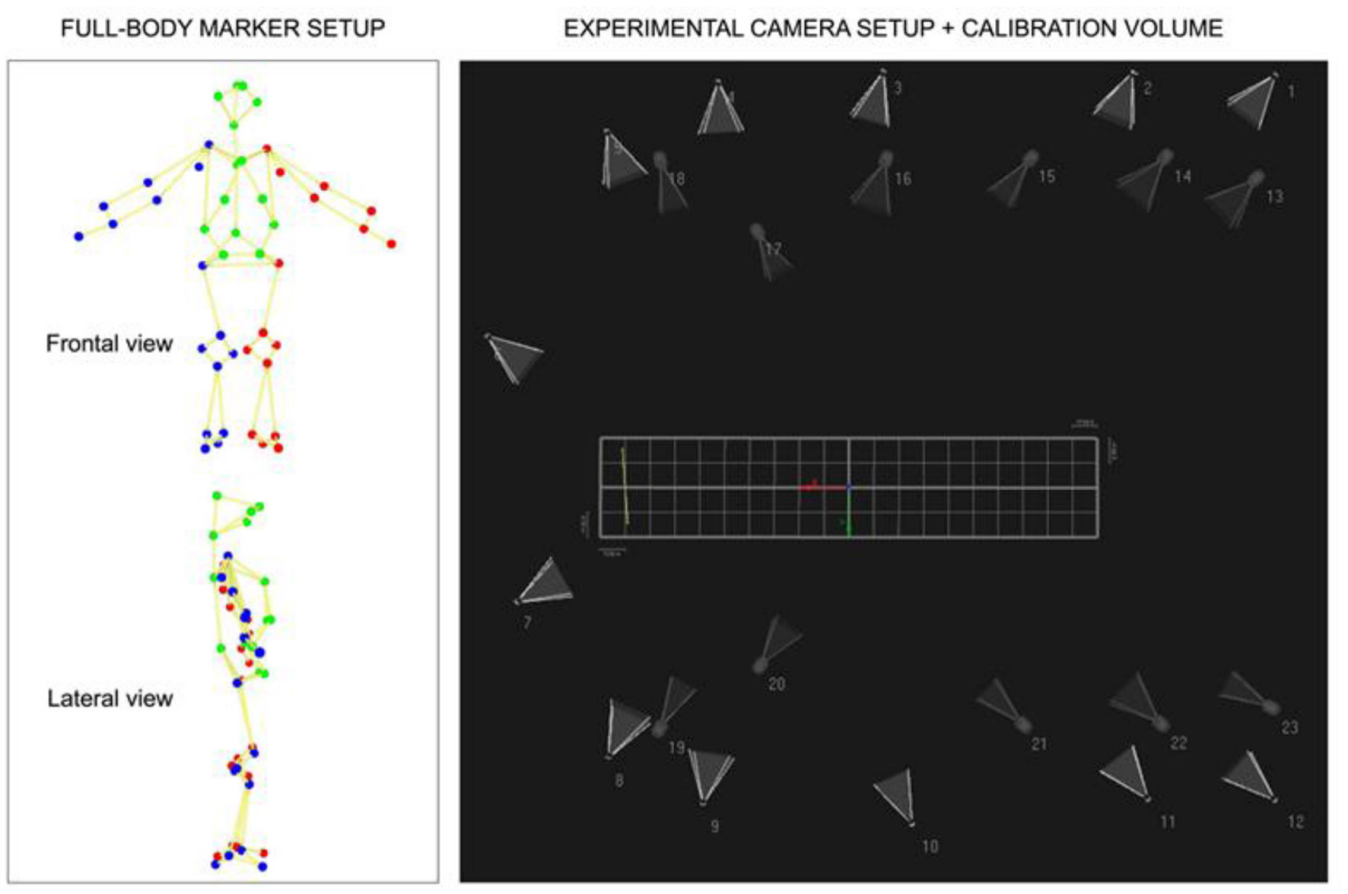
Configuration of kinematic-temporal data: full-body marker setup in Qualisys Track, experimental camera positioning with 12 land and 11 underwater cameras, and calibration volume covered. The orthogonal axes were defined as *X, Y*, and *Z* for horizontal, mediolateral, and vertical (*Z* = 0 defines water surface) movements. The yellow line depicts the reference system and positioning of the triaxial two force platforms.

Dual-media motion capture system with 12 land and 11 underwater cameras (Oqus 3 and 4 series, Qualisys, Gothenburg, Sweden) and a full-body marker setup (with 51 spherical retroreflective markers, see Figure 2) were used to track swimmer's actions at 100 Hz (Lauer et al., 2016) (see details of camera placement and configuration and calibration in Chainok et al., 2021). The kinetic assessment was obtained with two triaxial underwater force plates (Mourão et al., 2016) operating at a 2,000 Hz sampling frequency and fixed into the pool's wall on a custom built support (see details in de Jesus et al., 2019). The limits of this structure were identified with four retroreflective markers.

**Figure 3:**
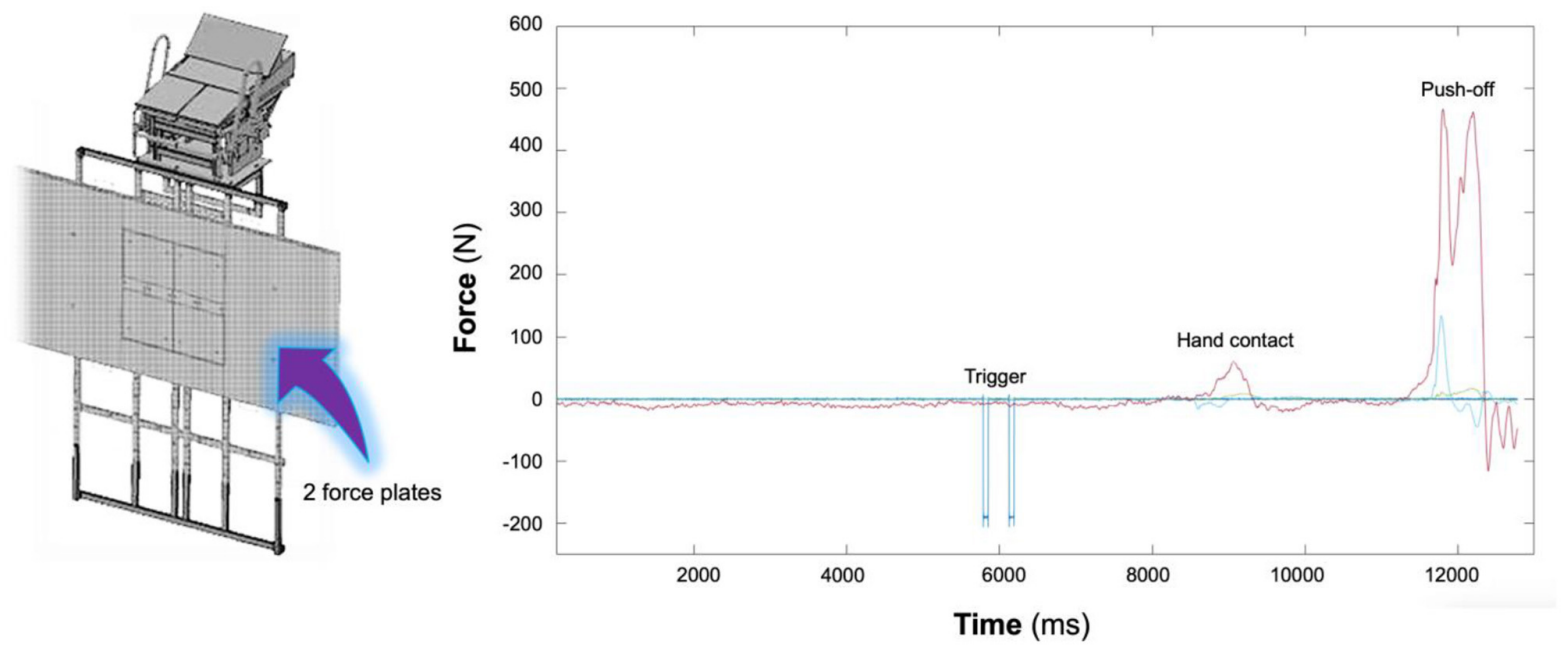
Kinetic data setup and data processing: two triaxial force plates set up and force-time curve of two triaxial force plate profiles (left and right panels). *Fx* and *Fy* are the mediolateral (green) and up and down (blue) components, and *Fz* is the horizontal force component (red).

**Figure 4:**
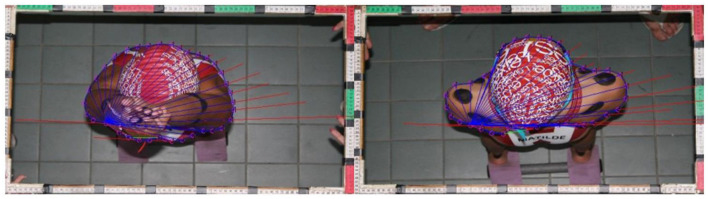
Body surface area determined through planimetry: data processing of the first and second gliding positions.

The 15 m turning time performance (composed of 7.5 m turn-in and 7.5 m turn-out of the wall segments) encompassed approaching, touching (wall contact), rolling, pushing glide, and swimming resumption until the vertex passes the 7.5 m marker (Figure 1). The Qualisys Track Manager (Oqus 3 and 4 series, Qualisys, Gothenburg, Sweden) software was used to acquire the temporal and 3D kinematic data. Built-in spline interpolation was used to fill markers' missing trajectories (representing up to ~50, 120, and 60 frames, i.e., 3.3, 8.0, and 4.0% of the trial duration in the approach, rotation, and turnout phases, respectively). The software Acqknowledge v.3.9.0 (BIOPAC Systems Incorporation, Santa Barbara, California, USA) was used to perform residual analysis to optimize the digital filter cutoff frequency (fast Fourier transform) and kinematic–temporal data were low-pass filtered using a digital filter with a cutoff frequency of 6 Hz (FIR—Window Blackman-61dB) (Acqknowledge, BIOPACiopac Systems Incorporation, Santa Barbara, California, USA). The bow wave effect at the beginning of the feet contact was considered negligible (not edited in the kinetic analysis) since swimmers glided in before touching the wall and rotated to push-off. Despite that, the underwater force platforms were synchronized with the motion capture system and the image-based kinematics allowed a reasonable verification of the force-to-time curve symmetry. Dominant (DPO) and non-dominant (NPO) push-off force terms were used to identify the characteristic peak force contributions in the *x, y*, and *z* components. Kinetic data processing was divided in to: (i) acquisition, plotting, and saving the strain readings of each triaxial force and the moment-of-force components from each force plate using a custom LabVIEW™ program (National Instruments, Austin, Texas, USA, http://www.ni.com/en-us/shop/labview.html) (Mourão et al., 2016; de Jesus et al., 2019); (ii) converting the strain readings into force values according to the previous calibration (Matlab R2014a, MathWork Inc., Natick, Massachusetts, USA), and (iii) filtering curves using a fourth-order zero-phase digital Butterworth filter with a 10 Hz cut-off frequency (Mourão et al., 2016; de Jesus et al., 2019) (Figure 3). The hydrodynamic variables (drag, drag coefficient, and body cross-sectional area) were assessed through an inverse dynamics approach (Vilas-Boas et al., 2010). We used planimetry (Clarys, 1979; Vilas-Boas et al., 2010) for cross-sectional area (S) assessment (Figure 4; see details in Chainok et al., 2021). The description of the studied kinematic-temporal, kinetic, and hydrodynamic variables is shown in Tables 1, 2.

**Table 1:** Kinematic-temporal variables selected for studying backstroke to breaststroke turning techniques.

**Variables**	**Definition**
7.5 m time-in (s)	Time between vertex reached 7.5 m wall distance at an origin of referential system until the hand wall touch.
5 m time-in (s)	Time between vertex reached 5 m wall distance at an origin of referential system until the hand wall touch.
2.5 m time-in (s)	Time between vertex reached 2.5 m wall distance at an origin of referential system until the hand wall touch.
Last upper limbs-wall distance (m)	Middle finger to wall distance at the last upper limbs cycle.
SL at last cycle (m)	The last upper limbs cycle length that was obtained by the horizontal displacement of the one upper limbs cycle.
Average SL during turn-in (m)	The mean of the last five upper limbs cycle length that was obtained by the horizontal displacement of the one upper limbs cycle.
Touching depth (m)	Depth at the hand beginning wall touch.
Hand contact time (s)	Time at hand wall contact.
Rotation time (s)	Time between hand contacts to feet contact.
Total wall contact time (s)	Total contact time of the feet with the wall.
Push-off time (s)	Time spent with the feet against the wall as the hips moved forward until the feet exited the wall.
Tuck index	The distance between the right hip and the wall at the start of the push-off is divided by the swimmer's lower limb.
Foot plant index	Depth of the wall foot plant at the beginning of push-off divided by swimmer's lower limb.
Push-off velocity (m·s^−1^)	Resultant velocity of sacrum at the feet had left the wall.
First gliding distance (m)	Distance of sacrum travel from the feet had left the wall to the first frame of transition phase.
First gliding time (s)	Time of sacrum travel from the beginning of feet had left the wall to the first frame of transition.
First gliding depth (m)	Mean of sacrum depth during the gliding phase.
Transition distance (m)	Distance of sacrum travel from the initial separation of the hands or starting dolphin kick until upper limbs fully extended at sides of the body.
Transition time (s)	Time of sacrum travel from the initial of hands separate or starting dolphin kick until the upper limbs fully extended at sides of the body.
Transition gliding depth (m)	Mean of sacrum depth during transition phase.
Second gliding distance (m)	Distance of sacrum travel from the first frame of the upper limbs fully extended at sides of the body to an instant which hands begins to move up from the body side.
Second gliding time (s)	Time of sacrum travel from the first frame of the upper limbs fully extended at the sides of the body to an instant which hands begins to move up from body side.
Second gliding depth (m)	Mean of sacrum depth during the second gliding phase.
Breakout distance (m)	Distance at which the head breaks the surface for the first time.
Breakout time (s)	Time from the feet had left the wall to the vertex breaks the surface for the first time.
Time-out (s)	Time from the feet had left the wall to the vertex reach 7.5 m mark.
15 m turn time (s)	The turn time performance including 7.5 m time-in and 7.5 m time-out.

**Table 2:** Kinetic and hydrodynamic variables selected for analyzing the backstroke to breaststroke turns.

**Variables**	**Definition**
Hand peak X force (N)	The highest force applied while hand pushing to the left or right on the force plate during hand contact.
Hand peak Y force (N)	The highest force applied while hand pushing up or down on the force plate during hand contact.
Hand peak Z force (N)	The highest force applied perpendicular to the force plate during hand contact.
Hand contact impulse (Z) (Ns.)	The area under the perpendicular Z force-time curve during hand contact.
Non-dominant peak_X push-off force: NPO_X (N)	The highest force applied while feet pushing to the left or right on the non-dominant force plate to the feet had left the wall.
Non-dominant peak_Y push-off force: NPO_Y (N)	The highest force applied while feet pushing up or down on the non-dominant force plate during to the feet had left the wall.
Non-dominant peak_Z push-off force: NPO_Z (N)	The highest force applied while feet horizontal pushing on the non-dominant force plate to the feet had left the wall.
Non-dominant push-off impulse (Z) (Ns)	The area under the Z force-time curve during the foot push-off non-dominant force plate to the feet had left the wall.
Dominant peak_X push-off force: DPO_X (N)	The highest force applied while feet pushing to the left or right on the dominant force plate to the feet had left the wall.
Dominant peak_Y push-off force: DPO_Y (N)	The highest force applied while feet pushing up or down on the dominant force plate during to the feet had left the wall.
Dominant peak_Z push-off force: DPO_Z (N)	The highest force applied while feet horizontal pushing on the dominant force plate to the feet had left the wall.
Dominant push-off impulse (Z) (Ns)	The area under the Z force-time curve during the foot push-off dominant force plate to the feet had left the wall.
First gliding drag force (N)	The passive drag force during the first gliding position that was assessed through inverse dynamics (*D* = *ma*).
Second gliding drag force (N)	The passive drag force during the second gliding position that was assessed through inverse dynamics (*D* = *ma*).
First gliding drag coefficient	The drag coefficient during the second gliding position that was assessed through inverse dynamics, following equation (*C_*D*_ = 2D/*ρ *S v^2^*).
Second gliding drag coefficient	The drag coefficient during the second gliding position that was assessed through inverse dynamics, following equation (*C_*D*_ = 2D/*ρ *S v^2^*).

### Statistical Analysis

Basic exploratory and descriptive statistics were computed using SPSS Statistics for Windows Version 24.0 (IBM Corporation, Armonk, New York, USA) aiming to detect potential errors in data entry and eventual outliers, and assessing data distribution normality (Shapiro–Wilk test), multicollinearity (variance inflation factors), and homoscedasticity (Levene's test). A one-way ANOVA was used to observe differences in the selected kinematic-temporal, kinetic, and hydrodynamic variables among the four different backstroke to breaststroke turning techniques. If a significant effect was found, *post-hoc* pairwise comparisons using Tukey's HSD were conducted. Then, factor analysis was conducted to lower the number of variables and to analyze the relationships structures between variables. For this purpose, selected variables were grouped into turn-in and turn-out variables (approach and rotation vs. wall contact, gliding, and pull-out phases), factors were chosen on the basis of a cutoff eigenvalue of 1, principal component extraction with a varimax rotation, and the scree plot proposed (Tor et al., 2015), and best-subsets analysis was conducted to determine the best regression equation for 15 m turn time prediction (using Minitab 19, Minitab Incorporation, State College, Pennsylvania, USA). Finally, a multiple regression analysis (with the enter method) was used to determine and predict the 15 m turn time based on each turning technique selected variables. The full multiple linear regression analysis was completed with SPSS based on the largest *R*^2^ value and the smallest error.

## Results

Descriptive- and variance-related analysis on selected variables for each backstroke to breaststroke turning technique is given in Table 3. The turning techniques showed no significant effects on the turn-in (F3, 232 = 0.61; *p* = 0.61), rotation time (F3, 232 = 0.69; *p* = 0.56), turn-out (F3, 232 = 0.33; *p* = 0.80), and 15 m turn times (F3, 232 = 0.64; *p* = 0.59).

**Table 3:** Descriptive- and variance-related statistics of the studied variables.

**Variables**	**Open**	**Somersault**	**Bucket**	**Crossover**	**Total**
7.5 m time-in (s)	7.42 ± 0.63	7.35 ± 0.55	7.30 ± 0.65	7.45 ± 0.70	7.38 ± 0.63
5.0 m time-in (s)	5.20 ± 0.54	5.15 ± 0.47	5.12 ± 0.59	5.21 ± 0.61	5.17 ± 0.55
2.5 m time-in (s)	2.48 ± 0.32	2.45 ± 0.29	2.52 ± 0.36	2.48 ± 0.34	2.48 ± 0.33
Last upper limbs-wall distance (m)	0.45 ± 0.25^s^	0.57 ± 0.25°	0.52 ± 0.25	0.48 ± 0.27	0.51 ± 0.26
SL at last cycle (m)	1.63 ± 0.28	1.55 ± 0.28	1.64 ± 0.31	1.63 ± 0.33	1.61 ± 0.30
Average SL during turn-in (m)	1.68 ± 0.20	1.65 ± 0.18	1.71 ± 0.21	1.70 ± 0.20	1.69 ± 0.20
Touching depth (m)	0.18 ± 0.09^s^	0.36 ± 0.13^°^^,^^b^^,^^c^	0.16 ± 0.09^s^	0.13 ± 0.06^s^	0.21 ± 0.13
Hand contact time (s)	0.49 ± 0.21^b^^,^^c^	0.49 ± 0.18^b^^,^^c^	0.59 ± 0.15^°^^,^^s^^,^^c^	0.37 ± 0.16^°^^,^^s^^,^^b^	0.48 ± 0.19
Hand peak X force (N)	1.59 ± 0.32	1.61 ± 0.23^c^	1.68 ± 0.26^c^	1.50 ± 0.23^s^^,^^b^	1.60 ± 0.27
Hand peak Y force (N)	8.56 ± 1.62^b^	8.48 ± 1.09^b^	17.37 ± 3.18^°^^,^^s^^,^^c^	9.05 ± 1.72^b^	10.78 ± 4.32
Hand peak Z force (N)	24.67 ± 29.47^s^	42.58 ± 51.80^°^^,^^c^	41.86 ± 52.89^c^	21.89 ± 26.11^s^^,^^b^	32.88 ± 12.85
Hand contact impulse (Z) (Ns.)	14.77 ± 3.19^s^^,^^b^	23.40 ± 4.41^°^^,^^c^	24.82 ± 5.03^°^^,^^c^	8.65 ± 1.53^s^^,^^b^	17.63 ± 7.25
Rotation time (s)	1.24 ± 0.18	1.28 ± 0.24	1.31 ± 0.27	1.33 ± 0.24	1.29 ± 0.23
Total wall contact time (s)	0.57 ± 0.19	0.54 ± 0.12^c^	0.53 ± 0.12^c^	0.63 ± 0.18^s^^,^^b^	0.57 ± 0.16
Push-off time (s)	0.38 ± 0.16	0.43 ± 0.13	0.37 ± 0.09^c^	0.46 ± 0.14^b^	0.41 ± 0.14
Tuck index	0.70 ± 0.15	0.75 ± 0.11	0.76 ± 0.10	0.72 ± 0.13	0.73 ± 0.13
Foot plant index	0.58 ± 0.19^s^^,^^c^	0.68 ± 0.19^°^^,^^b^^,^^c^	0.55 ± 0.18^s^	0.50 ± 0.15^°^^,^^s^	0.58 ± 0.19
Push-off velocity (m·*s*^−1^)	2.02 ± 0.31^c^	2.02 ± 0.33^c^	2.01 ± 0.29^c^	2.17 ± 0.37^°^^,^^s^^,^^b^	2.06 ± 0.33
First gliding distance (m)	2.40 ± 0.57	2.60 ± 0.69	2.50 ± 0.67	2.43 ± 0.69	2.47 ± 0.65
First gliding time (s)	1.21 ± 0.42	1.34 ± 0.52	1.31 ± 0.44	1.29 ± 0.41	1.28 ± 0.45
First gliding depth (m)	0.48 ± 0.09^s^^,^^b^	0.72 ± 0.15^°^^,^^b^^,^^c^	0.53 ± 0.14^°^^,^^s^	0.49 ± 0.13^s^	0.55 ± 0.16
Transition distance (s)	1.08 ± 0.20	1.08 ± 0.24	1.09 ± 0.16	1.10 ± 0.21	1.09 ± 0.20
Transition time (s)	0.98 ± 0.22	0.92 ± 0.20	0.96 ± 0.18	0.96 ± 0.19	0.96 ± 0.20
Transition gliding depth (m)	0.62 ± 0.15^s^	0.86 ± 0.18^°^^,^^b^^,^^c^	0.67 ± 0.20^s^	0.65 ± 0.17^s^	0.70 ± 0.20
Second gliding distance (m)	0.80 ± 0.24	0.86 ± 0.30	0.88 ± 0.28	0.88 ± 0.30	0.85 ± 0.28
Second gliding time (s)	0.77 ± 0.26	0.83 ± 0.36	0.86 ± 0.32	0.85 ± 0.35	0.82 ± 0.32
Second gliding depth (m)	0.61 ± 0.17^s^	0.76 ± 0.18^°^^,^^b^^,^^c^	0.62 ± 0.19^s^	0.62 ± 0.17^s^	0.65 ± 0.19
Breakout distance (m)	5.94 ± 0.86	6.12 ± 1.00	6.04 ± 0.93	6.02 ± 0.99	6.04 ± 0.94
Breakout time (s)	4.83 ± 0.95	4.99 ± 1.03	4.83 ± 0.97	4.79 ± 0.99	4.86 ± 0.98
Time-out (s)	7.30 ± 0.92	7.09 ± 0.97	7.07 ± 0.84	7.13 ± 0.89	7.12 ± 0.89
NPO_X (N)	1.64 ± 0.19	1.66 ± 0.22	1.67 ± 0.17	1.59 ± 0.24	1.64 ± 0.20
NPO_Y (N)	19.41 ± 8.25^s^^,^^c^	15.31 ± 8.35°	21.12 ± 10.07^c^	13.23 ± 5.42^°^^,^^b^	17.23 ± 8.65
NPO_Z (N)	49.36 ± 24.99	45.81 ± 37.63	36.37 ± 20.59	62.84 ± 44.57	48.96 ± 34.14
NPO_ Impulse (Z) (Ns)	34.02 ± 25.07^s^^,^^b^	21.91 ± 15.46^°^^,^^c^	17.56 ± 8.64^°^^,^^c^	31.14 ± 49.38^s^^,^^b^	26.70 ± 21.33
DPO_X (N)	21.66 ± 11.03^s^^,^^b^^,^^c^	12.99 ± 5.36°	14.74 ± 8.63^°^^,^^c^	8.03 ± 3.24^°^^,^^b^	14.64 ± 11.14
DPO_Y (N)	64.92 ± 37.27^s^	37.28 ± 20.18^°^^,^^b^^,^^c^	70.08 ± 43.10^s^	56.07 ± 27.76^s^	56.86 ± 35.05
DPO_Z (N)	145.45 ± 76.20^b^	140.090 ± 65.50^b^	194.41 ± 119.14^°^^,^^s^^,^^c^	141.44 ± 30.50^b^	153.65 ± 78.90
DPO_ Impulse (Z) (Ns)	53.07 ± 30.50^b^	52.03 ± 33.61^b^	57.75 ± 39.48^°^^,^^s^^,^^c^	49.92 ± 33.11^b^	53.04 ± 33.87
D_1_ (N)	−33.93 ± 7.56^c^	−36.40 ± 9.34^c^	−36.49 ± 5.39^c^	−40.57 ± 8.19^°^^,^^s^^,^^b^	−36.73 ± 8.32
D_2_ (N)	−62.70 ± 25.57	−62.86 ± 25.56	−63.27 ± 25.83	−67.29 ± 26.82	−62.59 ± 25.35
C_D1_	−0.74 ± 0.11	−0.72 ± 0.10	−0.75 ± 0.10	−0.76 ± 0.09	0.74 ± 0.10
C_D2_	−1.16 ± 0.38	−1.16 ± 0.37	−1.10 ± 0.27	−1.20 ± 0.38	−1.14 ± 0.36
15 m turn time (s)	16.53 ± 1.53	16.41 ± 1.47	16.27 ± 1.60	16.67 ± 1.52	16.48 ± 1.52

°, s, b, c *Significantly different from open, somersault, bucket and crossover turn (p < 0.05)*.

The best subsets regression for turn-in and turn-out to predict 15 m turning time in each backstroke to breaststroke turning technique are given in Tables 4, 5. Regarding the *open* turn, there were three predictors (7.5 m time-in, average SL, and hand contact time) explained 86% (*R*^2^ = 0.86; *p* < 0.01) for turn-in, five predictors (first gliding distance, first gliding time, second gliding depth, turn-out time, and DPO_Z) explained 93% (*R*^2^ = 0.93; *p* < 0.01) for turn-out on the 15 m turning time. For the *somersault* turn, there were three predictors (7.5 m time-in, 2.5 m time, and rotation time) explained 78% (*R*^2^ = 0.78; *p* < 0.01) for turn-in, seven predictors (wall contact time, first gliding distance, breakout distance, breakout time, turn-out time, DPO_Y, and C_D2_) explained 92% (*R*^2^ = 0.92; *p* < 0.01) for turn-out on the 15 m turning time, respectively.

**Table 4:** Data obtained from multiple regression analysis for turn-in variables.

**Turns**	**Variables**	***B***	***R***	***p***	**Full model**	
*Open* turn	Constant	4.49		0.01^**^	*R*	0.93
	7.5 m time-in	1.61	0.81	0.001^**^	*R^2^*	0.86
	Average SL	−1.00	−0.13	0.04^*^	*p*	0.001
	Hand contact time	−0.81	−0.11	0.04^*^		
	Equation: 15 m turn time = 4.49 + 1.61 × 7.5 m time-in – 1.00 × Average SL – 0.81 Hand contact time					
*Somersault* turn	Constant	2.26		0.04^*^	*R*	0.86
	7.5 m time-in	1.27	0.59	0.001^**^	*R^2^*	0.78
	2.5 m time-in	1.86	0.36	0.01^**^	*p*	0.001
	Last upper limbs -wall distance	−0.69	−0.11	0.11		
	Rotation time	−0.99	−0.16	0.03^*^		
	Equation: 15 m turn time =2.26 + 1.27 × 7.5 m time-in + 1.86 × 2.5 m time-in) – 0.99 × Rotation time					
*Bucket* turn	Constant	1.56		0.04^*^	*R*	0.95
	7.5 m time-in	1.45	0.73	0.001^**^	*R^2^*	0.89
	2.5 m time-in	0.94	0.23	0.03^*^	*p*	0.001
	Last upper limbs-wall distance	−0.76	−0.13	0.02^*^		
	Equation: 15 m turn time = 1.561 + 1.45 × 7.5 m time-in + 0.94 × 2.5 m time-in – 0.76 × Last upper limbs -wall distance					
*Crossover* turn	Constant	5.05		0.01^**^	*R*	0.93
	7.5 m time- in	2.21	1.18	0.001^**^	*R^2^*	0.87
	2.5 m time- in	−1.68	−0.36	0.03^*^	*p*	0.001
	Average SL	−1.23	−0.16	0.03^*^		
	Rotation time	−0.79	−0.14	0.02^*^		
	Equation: 15 m turn time =5.05 + 2.21 × 7.5 m time-in – 1.68 × 2.5 m time-in – 1.29 × average SL – 0.79 × rotation time					

*, ** *Significant for p < 0.05 and 0.01, respectively*.

**Table 5:** Data obtained from multiple regression analysis for turn-out variables.

**Turns**	**Variables**	***B***	***R***	***p***	**Full model**	
*Open*	Constant	−0.85		0.01	*R*	0.94
turn	Tuck index	−0.52	0.51	0.31	*R^2^*	0.93
	First gliding distance	1.01	0.34	0.01^**^	*p*	0.01
	First gliding time	−1.09	0.41	0.01^**^		
	Second gliding depth	−0.92	0.35	0.01^**^		
	7.5 m turn-out time	1.99	0.09	0.01^**^		
	NPO_X	0.52	0.29	0.08		
	NPO_Y	0.01	0.01	0.12		
	NPO_Impulse	−0.00	0.00	0.07		
	DPO_Z	0.01	0.00	0.01^**^		
	Equation: 15 m turn time = -0.85 + 1.01 × first gliding distance – 1.09 × first gliding time – 0.92 × second gliding depth + 1.99 × turn -out time + 0.01 × DPO_Z					
*Somersault*	Constant	8.44		0.001^**^	*R*	0.93
turn	Wall contact time	0.94	0.36	0.01^**^	*R^2^*	0.92
	Push-off velocity	0.37	0.20	0.08	*p*	0.01
	First gliding distance	0.33	0.15	0.04^*^		
	Breakout distance	−1.02	0.29	0.01^**^		
	Breakout time	0.61	0.22	0.01^**^		
	Turn out time	1.03	0.14	0.01^**^		
	DPO_Y	−0.01	0.00	0.01^**^		
	DPO_Z	−0.01	0.01	0.10		
	C_D2_	−0.72	0.17	0.10		
	Equation: 15 m turn time = 8.44 + 0.94 × wall contact time + 0.33 × first gliding distance – 1.02 × breakout distance + 0.61 × breakout time + 1.03 × turn-out time – 0.01 × DPO_Y – 0.72 × C_D2_					
*Bucket*	Constant	5.28		0.01^**^	*R*	0.94
turn	Foot plant index	−0.78	0.35	0.03^*^	*R^2^*	0.92
	Push-off time	1.30	0.78	0.10	*p*	0.01
	Push-off velocity	−0.47	0.24	0.04^*^		
	Second gliding distance	−0.75	0.27	0.01^**^		
	Turn -out time	1.47	0.09	0.01^**^		
	DPO_X	0.01	0.01	0.07		
	C_D1_	0.43	0.19	0.03^*^		
	Equation: 15 m turn time = 5.28 – 0.78 × foot plant index – 0.47 × push – off velocity – 0.75 × second gliding distance + 1.47 × turn -out time + 0.43 × C_D1_					
*Crossover*	Constant	5.35		0.01^**^	*R*	0.92
turn	Tuck index	−1.06	0.64	0.11	*R^2^*	0.90
	Push-off velocity	−0.46	0.25	0.07	*p*	0.01
	Breakout time	−0.18	0.09	0.04^*^		
	Turn-out time	1.74	0.11	0.01^**^		
	NPO_Y	−0.05	0.02	0.01^**^		
	NPO_Z	0.01	0.00	0.01^**^		
	D_1_	−0.01	0.01	0.02^*^		
	D_2_	−0.01	0.00	0.03^*^		
	C_D2_	0.76	0.28	0.01^**^		
	Equation: 15 m turn time = 5.35 – 0.18 × breakout time + 1.74 × turn-out time – 0.05 × NPO_Y + 0.01 × NPO_Z – 0.01 × D_1_ – 0.01 × D_2_ + 0.76 × C_D2_					

*, ** *Significant for p < 0.05 and 0.01, respectively*.

For the *bucket* turn, there were three predictors (7.5 m time-in, 2.5 m time-in, and last upper limbs-wall distance) explained 89% (*R*^2^ = 0.89; *p* < 0.01) for turn-in, five predictors (foot plant index, push-off velocity, second gliding distance, turn-out time, and C_D1_) explained 92% (*R*^2^ = 0.92; *p* < 0.01) for turn-out on the 15 m turning time. For the *crossover* turn, there were four predictors (7.5 m time-in, 2.5 m time-in, average SL, and rotation time) explained 87% (*R*^2^ = 0.87; *p* < 0.01) for turn-in, seven predictors (breakout time, turn-out time, NPO_Y, NPO_Z, D_1_, D_2_, and C_D2_) explained 90% (*R*^2^ = 0.90; *p* < 0.01) for turn-out on the 15 m turning time, respectively.

## Discussion

The main aim of this study was to identify the biomechanical features of backstroke to breaststroke transition techniques (*open, somersault, bucket*, and *crossover*) in age-group swimmers. We believed that 15 m turning time performance is described by combining contributions from the turn-in and turn-out phases, and different combinations of feature variables depending on the chosen backstroke to breaststroke transition technique. As expected, general turn-in performance can be predicted mostly by faster times during the 7.5 m, 2.5 m to the wall. The average SL is a predictor of turn-in performance for both open and crossover turns, with faster rotation time being the most relevant variable for somersault and crossover turns. The last upper limbs-to-wall distance, which refers to kinesthetic awareness and sense of space, affects bucket turn performance. Our results from the turn-out phase highlighted the importance of the interaction between kinematic and kinetic variables at the wall contact and push-off phase, which influenced turn-out performance across all backstroke to breaststroke turns studied. However, the importance of the turn-out variables changes depending on the chosen technique.

### *Open* Turn

The 7.5 m time-in, average stroke length, and hand contact time were the three key variables for the turn-in performance, while the first gliding distance, first gliding time, second gliding depth, turn-out time, and dominant push-off_Z force were identified as key for the turn-out. Our results are consistent with some previous findings in elite swimmers that indicated that their turn-in performance was highly associated with their total turn time in the 200 and 400 m backstroke to breaststroke (Mason and Cossor, 2001). From the perspective of turn-in performance, the simple direction switch from the supine to the prone position during the *open* turn may require specific skills to maintain the swimming speed that incorporates the fastest rotation or pivot execution (Blanksby et al., 2004; Webster et al., 2011).

It has been reported that the optimization of the relationships between the kinematic, kinetic, and hydrodynamic variables can directly influence turn-out performance (Termin and Pendergast, 1998; Vilas-Boas et al., 2010; Pereira et al., 2015). The *open* turn turn-out performance mainly depends on the interaction between the kinetic variable (dominant push-off_Z force) and the four kinematic-temporal variables (first gliding distance, first gliding time, second gliding depth, and turn-out time). Theoretically, the peak perpendicular force, total impulse, and wall contact time kinetic features are key factors of swimming turns (Prins and Patz, 2006), with the dominant peak push-off_Z force being the key kinetic variable in this study. It tended to be slightly lower than data previously obtained in the breaststroke (557 ± 109 N; Blanksby et al., 1998), rollover backstroke (229 ± 70 N; Blanksby et al., 2004), and tumble turns (693 ± 228 N; Blanksby et al., 1998) in age-group swimmers. However, this is not particularly surprising considering that the age-group swimmers from our study depicted a slower rotation with a tendency to spend a short preparatory push-off time (33%), which could lead to a lower maximum normalized peak force and impulse.

From the perspective of turn-out efficacy, the optimization of the underwater gliding depth, gliding time, and gliding distance will directly affect turning performance (Termin and Pendergast, 1998; Chainok et al., 2021). The first gliding distance and time, second gliding depth, and turn-out time were identified as key variables and appeared to be advantageous for performing an *open* turn. In the current study, the first and second gliding distances, and the breakout distance and time, were slightly shorter in the *open turn* than in the other three turns.

### *Somersault* Turn

The key mechanical features of the turn-in phase of the *somersault* turn mainly depended on the time-in (7.5 and 2.5 m) and rotation time. Given the high impact of the turn-in phase on the 15 m turning performance, the swimming approach (7.5 m and 2.5 m turn-in times), and rotation times should be more deeply considered. The *somersault* turn, compared to the *open* turn findings, suggests that a faster approach could directly influence the turn time. Since the execution of the *somersault* turn requires a hand touch at the wall before rotating from the supine to the prone position, the rotation is critical. At this backstroke to breaststroke transition, the rotation time tended to be slightly slower than those previously studied in the rollover backstroke (Blanksby et al., 2004) and breaststroke turns (Blanksby et al., 1998) by age-group swimmers.

The analysis of the turn-out variables revealed that the wall contact time, first gliding distance, breakout distance, breakout time, and turn-out time (kinematic-temporal), dominant push-off peak_Y force (kinetic and C_D2_ (hydrodynamic) variables were those affecting the 15 m turn time. Based on the pull-out strategy evidence, breakout distance, breakout time, and turn-out time were identified as the important variables, indicating that age-group swimmers should select their own individual strategies by considering the breakout distance and the time to maximize the pull-out performance (Blanksby et al., 2004). The longer first gliding distance in *somersault* turn may be related to a lower dominant peak push-off_Y coupled with a deeper foot plant, suggesting that age-group swimmers should try to minimize the up or down movement of the all body during push-off, which could lead to a longer and deeper gliding (Blanksby et al., 2004).

Contrary to the expectations, the dominant peak push-off_Y force (about 26% of the mean peak_Z force) was selected as a critical predictor of the 15 m turn time. Theoretically, the push-off force with the feet pushing up or down directly affects the push-off velocity and tends to be inversely related with rollover time (Blanksby et al., 2004; Pereira et al., 2015). The evidence from this study points to the notion that a suitable feet push-off position and wall contact time can directly affect the performance of the subsequent horizontal push-off force and impulse (Blanksby et al., 2004), and the push-off velocity (Pereira et al., 2015).

In the discussion of turn-out performance, it is essential to consider swimmers' hydrodynamic characteristics and pull-out strategy (Chainok et al., 2021). In the *somersault* turn, push-off from the wall that is completely ventral and without any relevant rotation of the body may eventually lead to lower hydrodynamic drag (Pereira et al., 2015). The current study C_D2_ of the *somersault* turn was slightly high, probably due to the lower foot plant index during the push-off phase that might directly affect the gliding path adopted during the pull-out phase (see Table 3). Even so, this value tended to be higher than those obtained in national-level breaststrokers (0.61–0.72; Vilas-Boas et al., 2010) and similar to data determined by computational fluid dynamics (0.85–1.06; Marinho et al., 2011).

### *Bucket* Turn

Multiple linear regression analysis indicated that optimal turn-in performance mainly depends on the 7.5 and 2.5 m times-in and last upper limbs-wall distance. There was a direct relationship between 15 m turn time and 7.5 m time-in (*r* = 0.93) and 2.5 m time-in (*r* = 0.85), and a small inverse relationship between 15 m turn time and last upper limbs-wall distance (*r* = −0.13). As in the *open* and *somersault* turns, speed-in was an essential influencing factor of turning performance, in agreement with the previous literature on elite (Nicol et al., 2019) and Olympic swimmers (Mason and Cossor, 2001). The last upper limbs-wall distance was similar among the four turning techniques (range 0.45–0.57 m), evidencing a tendency for consistency in the approaching speed, resulting in an optimal last upper limbs wall distance and leading to faster turn-in.

The foot plant index, push-off velocity, second gliding distance, and turn-out time (kinematic-temporal) and C_D1_ (hydrodynamic) variables were identified as the key variables for the backstroke to breaststroke turning performance. From the perspective of push-off efficacy, it is advantageous to address the appropriate lower extremity at wall contact with a greater tuck index and optimal feet planting (30–40 cm depth), which will facilitate the best horizontal push-off velocity (Clothier et al., 2000; Prins and Patz, 2006). However, the turning technique showed no main effect on push-off velocity and the linking and interaction of the kinematic variables at the wall contact and push-off phase can be considered a partial contribution of the biomechanical variables to turning performance. In the current study, the tuck index and, concomitant with a longer wall contact time tended to be higher than those for the butterfly turn (0.56 ± 0.11 s and 0.37 ± 0.09 s; Ling et al., 2004) and for the breaststroke turn (0.58 ± 0.13 s and 0.39 ± 0.08 s; Blanksby et al., 1998), performed by age-group swimmers. The foot plant index (0.55 ± 0.18) was also higher than the one previously obtained in flip turn performed by university swimmers (0.45 ± 0.10; Prins and Patz, 2006).

As determined before using inverse dynamics, the first gliding position at the breaststroke underwater path was more hydrodynamic than the second one, allowing lower S, C_D_, and D values for the same range of speeds (Vilas-Boas et al., 2010). The C_D1_ calculated in the *bucket* turn tended to be higher than that calculated in national-level breaststrokers (0.46 ± 0.08; Vilas-Boas et al., 2010), probably due to the lower gliding velocity and anthropometric characteristics of our age-group swimmers. Our data and the available literature also suggest that age-group swimmers need to be concerned about minimizing hydrodynamic drag by controlling their gliding position (body shape and length) along with their optimal gliding depth (range 0.4–0.6 m) (Lyttle et al., 2000; Vilas-Boas et al., 2010; Chainok et al., 2021).

### *Crossover* Turn

We have observed that the optimal *crossover* turn-in performance can be identified by the 7.5 and 2.5 m times-in, average stroke length, and rotation time, with the first two variables displaying strong direct relationships with 15 m turn time and the mean stroke length relating inversely with the 15 m turn time. Notably, the turn-in time and the wall approach stroke length were the key variables in all the backstroke to breaststroke turning techniques, indicating that the wall approach strategy was consistent among them.

Theoretically, from the turn-in efficacy improvement perspective, it is important to maximize the approach speed and minimize the rotation time. In the current study, the turning technique had no main effect on rotation time, which came out as a surprise because, from a theoretical and technical perspective, differences in body rotation actions—which are characteristic of the different studied techniques, may directly affect rotation speed and turning performance. Interestingly, the implemented training program significantly improved rotation in all the backstroke to breaststroke turning techniques, inclusively with higher values than those previously presented for the rollover backstroke (0.70 ± 0.10 s; Blanksby et al., 2004), pivot breaststroke (1.15 ± 0.22 s; Blanksby et al., 1998), pivot butterfly (1.11 ± 0.18 s; Ling et al., 2004), and tumble freestyle turns (2.01 m·s^−1^; Blanksby et al., 1996) performed by age-group swimmers.

Multiple linear regression analysis indicated that the breakout and turn out times, non-dominant peak push-off_Y and Z forces, and D_1_, D_2_, and C_D2_ are turn-out performance determinants and, due to the high impact of maximized breakout distance and streamlined position on the turn-out performance, the importance of those hydrodynamic variables should be emphasized. In fact, minimizing the hydrodynamic drag should be the primary consideration for improving backstroke to breaststroke turn-out performance. Typically, the first gliding position is more hydrodynamic than the second one, allowing lower S, D, and C_D_ values for the same range of speeds (Vilas-Boas et al., 2010; Marinho et al., 2011; Chainok et al., 2021). The *crossover* turn had g higher D_1_, D_2_, and C_D2_ values than the other studied turns, which may be justified by: (i) a worst streamline performance due to the lateral body movements that occur from the wall push-off to the first gliding position may (Lyttle et al., 1998; Termin and Pendergast, 1998) and (ii) the lower gliding velocity and control of the body shape and length while gliding. The current study *Crossover* D_1_, D_2_, and C_D2_ values were also slightly higher than previous values obtained in national-level swimmers (Vilas-Boas et al., 2010).

Our push-off force results are consistent with Araujo et al. (2010) findings indicating the highest normalized horizontal peak force contributes the most to enhancing turning performance in freestyle flip turns performed by national and international level swimmers while increasing the upward or downward wall push-off was found to have a negative impact on turn-out performance during rollover backstroke turn in age-group swimmers (Blanksby et al., 2004). Interestingly, the non-dominant Y and Z push-off forces play a critical role in determining the symmetry of lower limb push-off and subsequent gliding orientation. This finding implies that the *crossover*, in which the swimmer lateral push-off against the wall, may need a powerful extension of one of the lower limbs—possibly the dominant limb—to generate a symmetric push-off force.

## Conclusion

The determinant variables of the different backstroke to breaststroke transition techniques change during both the turn-in and turn-out phases. Some kinematic-temporal variables are more relevant during turn-in, some kinetic variables gain relevance during turn-out (highlighting the importance of the push-off phase), and the hydrodynamic variables are important for all the studied transition techniques. Finally, the rotation and push-off phases were the stronger determinants of turning performance among all studied backstroke to breaststroke turns. Considering the key biomechanical variables that influence each turning performance in the current data, the development of a specific training program aiming to enhance turning skills, particularly focusing on the rotation and push-off phases, should be reconsidered by coaches who work with age-group swimmers, even if it implies in a longer training intervention.

## Data Availability Statement

The raw data supporting the conclusions of this article will be made available by the authors, without undue reservation.

## Ethics Statement

The studies involving human participants were reviewed and approved by Faculty of Sport, University of Porto. Written informed consent to participate in this study was provided by the participants' legal guardian/next of kin.

## Author Contributions

PC, RZ, RF, and JV-B: conceptualization, methodology, writing—original draft preparation, and project administration. PC, KJ, RZ, LM, and PF: data curation. PC, KJ, LM, PF, RZ, RF, and JV-B: review and editing. PC and RZ: visualization. JV-B: supervision. All the authors have read and agreed to the published version of the manuscript.

## Funding

This study was supported by the Faculty of the Sport Science, Burapha University, Thailand (grant number 062/2554). RZ was founded by Research Center in Physical Activity, Health and Leisure—CIAFEL—Faculty of Sports, University of Porto—FADEUP (FCT UID/DTP/00617/2020 and Laboratory for Integrative and Translational Research in Population Health (ITR), Porto, Portugal (LA/P/0064/2020).

## Conflict of Interest

The authors declare that the research was conducted in the absence of any commercial or financial relationships that could be construed as a potential conflict of interest.

## Publisher's Note

All claims expressed in this article are solely those of the authors and do not necessarily represent those of their affiliated organizations, or those of the publisher, the editors and the reviewers. Any product that may be evaluated in this article, or claim that may be made by its manufacturer, is not guaranteed or endorsed by the publisher.
